# The Impact of Bio-Stimulants on Cd-Stressed Wheat (*Triticum aestivum* L.): Insights Into Growth, Chlorophyll Fluorescence, Cd Accumulation, and Osmolyte Regulation

**DOI:** 10.3389/fpls.2022.850567

**Published:** 2022-02-18

**Authors:** Fozia Farhat, Muhammad Arfan, Xiukang Wang, Arneeb Tariq, Muhammad Kamran, Hafiza Naila Tabassum, Ifra Tariq, Freddy Mora-Poblete, Rashid Iqbal, Ahmed M. El-Sabrout, Hosam O. Elansary

**Affiliations:** ^1^Department of Botany, University of Agriculture (UAF), Faisalabad, Pakistan; ^2^Department of Botany, Government College Women University, Faisalabad, Pakistan; ^3^Shaanxi Key Laboratory of Chinese Jujube, College of Life Sciences, Yan’an University, Yan’an, China; ^4^School of Agriculture, Food and Wine, The University of Adelaide, Adelaide, SA, Australia; ^5^Institute of Home and Food Sciences, Government College University Faisalabad, Faisalabad, Pakistan; ^6^Institute of Biological Sciences, University of Talca, Talca, Chile; ^7^Department of Agronomy, Faculty of Agriculture and Environment, The Islamia University of Bahawalpur, Bahawalpur, Pakistan; ^8^Department of Applied Entomology and Zoology, Faculty of Agriculture (EL-Shatby), Alexandria University, Alexandria, Egypt; ^9^Plant Production Department, College of Food & Agriculture Sciences, King Saud University, Riyadh, Saudi Arabia

**Keywords:** gaseous exchange rate, secondary metabolites, Cd accumulation, ascorbic acid, moringa leaf extract, growth, chlorophyll fluorescence

## Abstract

It has been established that wheat (*Triticum aestivum* L.) has a higher Cd absorption capacity than other cereal crops causing an excess daily Cd intake and a huge threat for public health. Therefore, the reduction of Cd accumulation in wheat from the soil is a crucial food-security issue. A pot trial was performed on Cd-stressed wheat seedlings to evaluate the morphological and physio-biochemical responses *via* foliage spray of two different bio-stimulants, i.e., ascorbic acid (AsA) and moringa leaf extract (MLE). Two wheat cultivars (Fsd-08 and Glxy-13) were exposed to cadmium (CdCl_2_.5H_2_O) stress (0, 500, and 1,000 μM), along with foliar spray of AsA (0 and 50 mM) and MLE (0 and 3%). The most observable growth reduction was documented in plants that are exposed to a higher Cd concentration (1,000 μM), followed by the lower Cd level (500 μM). The wheat growth attributes, such as number of leaves per plant, number of tillers per plant, biomass yield, shoot/root length, and leaf area, were greatly depressed under the Cd stress, irrespective of the cultivar. Under the increasing Cd stress, a significant diminution was observed in maximum photochemical efficiency (Fv/Fm), photochemical quenching (qP), and electron transport rate (ETR) accompanied with reduced gas exchange attributes. However, Cd-induced phytotoxicity enhanced the non-photochemical quenching (NPQ) and internal carbon dioxide concentration (Ci), which was confirmed by their significant positive correlation with Cd contents in shoot and root tissues of both cultivars. The contents of proline, AsA, glycine betaine (GB), tocopherol, total free amino acid (TFAA), and total soluble sugar (TSS) were greatly decreased with Cd stress (1,000 μM), while MLE and AsA significantly enhanced the osmolytes accumulation under both Cd levels (especially 500 μM level). The Cd accumulation was predominantly found in the root as compared to shoots in both cultivars, which has declined after the application of MLE and AsA. Conclusively, MLE was found to be more effective to mitigate Cd-induced phytotoxicity up to 500 μM Cd concentration, compared with the AsA amendment.

## Introduction

Heavy metal toxicity in the rhizosphere is a potential health menace to the growing population, as well as great anxiety for environmentalists globally (Ali et al., 2013; Sabir et al., 2020; Turan, 2021a). Cadmium (Cd) is generally released from industries due to various anthropogenic activities, excessive utilization of fertilizers (especially phosphate) in agricultural soils, and natural weathering of rocks and minerals (Ahanger et al., 2017; Turan et al., 2018; Wang et al., 2021; Xing et al., 2022). The rapid increase in industrialization, urbanization, and improper environmental planning has caused various limitations including severe yield reduction in various crop plants such as *Zea mays* L. (An et al., 2022), *Oryza sativa* L. (Sun et al., 2021), and *Triticum aestivum* L. (Hao et al., 2021).

It has been well-established that wheat (*Triticum aestivum* L.) has a higher Cd absorption capacity than other cereal crops (Zhou et al., 2021). In developing countries, more than 80% of wheat supply is used as a staple food, compared to developed countries that use less than 50% (Awika, 2011); thus, inhabitants may intake a high quantity of Cd through grains posing a high health risk to human beings (Abedi and Mojiri, 2020). Previous researchers found that the Cd in wheat is taken up through roots and then translocated to the aerial parts, thus, interfering with major metabolic processes to hinder normal growth and reproduction activities (Abedi and Mojiri, 2020). The photosynthetic processes are the chief modulators for the growth and development of plants (Maishanu et al., 2017). An elevated level of Cd in the rhizosphere imposed severe phytotoxic effects in plant tissues (Riaz et al., 2021b), which replicate itself by hampering plant’s morphological, physiological, and biochemical processes (Wali et al., 2015), like destroying the leaf chlorophyll structure to deteriorate photosynthetic and gas exchange relations (Vieira Santos et al., 2001; Malik et al., 2021). The photosynthetic system is highly prone to any modulation in the surrounding environment. Any environmental constraints will directly damage the structural and functional capacities of the photosynthetic apparatus and may disturb plant growth and survival (Saleem et al., 2020).

It has been established that Cd induces toxic effects by reacting with sulfur and nitrogen atoms (Astolfi et al., 2004). These atoms are involved in the biosynthesis of certain important amino acids that in turn, inhibit the biosynthesis of vital osmolytes in crop plants (Dong et al., 2016). The increased accumulation of Cd in various plant tissue contributes toward the enhanced generation of free radical oxygen species, which induce oxidative stress in plants (Gill et al., 2012; Mwamba et al., 2016a,b; Riaz et al., 2021a). These radical oxidative species also destabilize plant development by distorting various signal transduction pathways like antioxidant systems, photosynthesis, and hormonal signaling in different manners. The inception of such stress-stimulated responses is to counteract the injury and develop a system of their survivability under adverse conditions (Noctor et al., 2018). Plants, in response to major abiotic threats, try to alleviate stress and enhance their physiological and cellular functioning through their innate mechanism (Ali et al., 2020b; Riaz et al., 2021b). Accumulating osmolytes, or compatible solutes, is one of the major strategies to avoid damage to cellular machinery and to interfere with normal metabolic processes (Fujita et al., 2006).

Natural bio-stimulants are the most promising and suitable strategy nowadays to address yield losses caused by various stresses, which are intensified by climate change. Biostimulants possess many diverse compounds, with positive effects in plants to increase growth, diminish stress-induced restrictions, and enhance yield (Yakhin et al., 2017). The moringa (*Moringa oleifera*) leaf extract (MLE) has been categorized as one of the novel and natural biostimulants that are enriched with higher nutrient contents, improved growth, and Cd tolerance in *Phaseolus vulgaris* L. (Howladar, 2014), *Zea mays* L. (Basra et al., 2011; Bashir et al., 2021), *Lepidium sativum* L. (Khalofah et al., 2020), and *Triticum aestivum* L. (Fozia et al., 2021). The MLE is a prolific source of antioxidants, plant growth-promoting substances, and macro- and micronutrients (Khan et al., 2017), which are critical for the regulation of physiological, biochemical, and yield parameters of plants that are under stressed and normal environmental conditions (Rady et al., 2013; Khan et al., 2021; Rashid et al., 2021). Ascorbic acid (AsA), which is a commonly found non-enzymatic antioxidant, has significant potential for scavenging reactive oxygen species (ROS) and regulates several biochemical processes of considerable importance both under stressed and normal conditions (Ali et al., 2020a; Parveen et al., 2020). Previously, it was reported that exogenous application of AsA could significantly increase endogenous production of AsA in wheat tissues which might be involved in the biosynthesis of some vital molecules to trigger a powerful response against Cd stress (Jung et al., 2020). Further, the AsA applications have been observed to improve the various physiological and biochemical processes in barley (*Hordeum vulgare* L.) (Yaseen et al., 2021) and wheat (*Triticum aestivum* L.) (Al-Hakimi and Hamada, 2011) grown under Cd stress. Therefore, it is interesting to compare the costs and benefits of using pure organic active compounds to increase yield under abiotic stress conditions (García-García et al., 2020).

The beneficial role of the combined application of MLE and AsA to mitigate Cd stress in the wheat plant is scarcely available. Therefore, the current study was based on the hypothesis that MLE and AsA will enhance the tolerance of wheat seedlings by maintaining morpho-physiological responses and accumulation of secondary metabolites to reduce the impact of Cd. Our findings will provide an insight to guide further studies of the Cd resistance with MLE in wheat at the molecular level, as well as tolerance against other heavy metals in other plant species. The objectives of the current study were to: (i) investigate the impact of Cd on wheat growth attributes, chlorophyll fluorescence, antioxidant, and osmolyte performance, and (ii) explore valuable physiological and biochemical traits as a marker to induce Cd tolerance with exogenously applied MLE and AsA. The findings would further highlight the mechanisms involved to ensure tolerance against Cd in wheat seedlings, along with the provision of useful, innovative, and cheap techniques in the form of moringa to mitigate other abiotic stresses.

## Materials and Methods

### Materials and Growth Environment

A pot investigation was performed in the wirehouse of the old botanical garden, University of Agricultural, Faisalabad, Pakistan (31.26” latitude, 73.06” longitude). The growing conditions in the wirehouse remained at 27/20°C day/night temperature, a 12-h illumination per day, and 65–70% moisture contents. The seeds of two wheat cultivars, i.e., Faisalabad-08 (Fsd-08) and Galaxy-13 (Glxy-13), were provided by Ayyub Agriculture Research Institute (AARI), Faisalabad, Pakistan. Healthy seeds were surface-sterilized by soaking in the solution of sodium hypochlorite for 5 min and washed with distilled water repeatedly. The plastic pots (32 × 22 cm) were filled with 5.5 kg dried and sieved sand. Later, the dried seeds were sown in pots for germination. All the pots were watered with a half-strength Hoagland nutrient medium once a week.

### Experimental Design

Both wheat cultivars were allowed to grow up to the three-leaf stage, thinning was done to ensure 5 uniform seedlings/pot before application of Cd, ascorbic acid (50 mM), and moringa leaf extract (3%). The Cd was saturated in the rhizosphere by dissolving CdCl_2_.5H_2_O in a full-strength Hoagland nutrient medium (Hoagland and Arnon, 1950), while AsA and MLE were applied as foliar treatment having 2 ml between -20 in each solution. The stock solution of Cd was prepared in mmol/L (mM), which was further diluted to micromole/L (μM) to maintain 500 and 1,000 μM Cd concentrations. Seven treatments were laid down in completely randomized design, and designated as Control, Cd_500_, Cd_1000_, Cd_500_ + AsA, Cd_500_ + MLE, Cd_1000_ + AsA, and Cd_1000_ + MLE, respectively. Moringa leaves were obtained from the forestry department, University of Agriculture, Faisalabad and authenticated by the local experts. Leaves were frozen at -80°C for 72 h and extract was made in a locally fabricated extract machine with water (10:1; fresh leaves: water). The concentrated extract was further diluted to 3% with distilled water before foliar application. To maintain the moisture level of plants up to 75–85%, pots were watered regularly, while Cd and foliar application were given once a week (6 weeks). To avoid nutrient deficiency, the nutrient solution was irrigated once a week as well. All the below-mentioned attributes were recorded 45 days after sowing (DAS), with and without treatment. All the measurements were performed according to the set criterion to reduce the difference caused by determination time.

### Determination of Morphological and Gas Exchange Attributes

The shoot and root length and the fresh biomass were measured separately with the measuring tape (cm) after harvesting and subsequent weighing, respectively. Leaf area (cm^2^) was calculated by taking the length and width of three randomly selected leaves/plant/replicate with a measuring tape. The number of tillers and the number of leaves were recorded by counting the respective trait.

The gaseous attributes, such as carbon dioxide assimilation rate (*A*), stomatal conductance (*Gs*), internal CO_2_ concentration (*Ci*,), and transpiration rate (E), were assessed using fully turgid uppermost leaves with a portable photosynthesis system, Infra-Red Gas Analyzer (LCA-4 ACD, Analytical Development, Hoddesdon, United Kingdom) at a light-saturating intensity between 9:00 am and 12:00 pm. On the other hand, water-use efficiency was calculated by adopting the formula (*A/E*).

### Determination of Photosynthetic Pigments and Chlorophyll Fluorescence Parameters

The concentrations of chlorophyll a or b, total chlorophyll, and carotenoid contents (mg g^–1^ FW) were examined by following the method devised by Arnon (1949). In brief, about.25 g of fresh leaf discs (45 days old) were dipped in 5 ml of 80% acetone solution. The mixture was placed in a dark place overnight. After overnight extraction, the optical density (OD) of the acetone extract was measured at 663, 645, and 480 nm. The obtained OD values were used to calculate chlorophyll a or b, total chlorophyll contents (*a* + *b*), and carotenoids by applying the respective formula.

The chlorophyll fluorescence (CF) parameters from the upper leaf surfaces of wheat seedlings were determined with an OS5p Modulator Fluorometer [ADC BioScientific Ltd., Great Amwell Herts, United Kingdom (Strasserf and Srivastava, 1995)]. Three seedlings from each treatment were randomly chosen and used to measure the CF parameters. After 30 min of the dark adaptation, the minimal fluorescence (F_0_) was determined by a weak red light (< 0.1 μmol m^–2^ s^–1^). Then, the maximum fluorescence (Fm) was determined using a saturating pulse (8,000 μmol m^–2^ s^–1^) of.8 -s duration. The activity of photosystem II (PSII) was determined by *F_*V*_/F_*M*_*, when the leaves had been dark-adapted for 30-min, using the dark leaf clip. Quantum efficiency of photochemical transports used for photosynthesis (Φ*_*PSII*_*), photochemical quenching (qP), quantum efficiency of thermal dissipation promoted by the photoprotective non-photochemical quenching *via* the xanthophyll cycle (Φ*_*NPQ*_*), and electron transport rate (Φ*_*ETR*_*) was calculated using 1,500 μmol m^–2^ s^–1^ actinic light.

### Determination of Glycine Betaine, Ascorbic Acid, Proline, and Tocopherol Contents

For GB, a slightly modified method of Grieve and Grattan (1983) was adopted. Dry leaves (0.25 g) were extracted with.5% toluene. Extracts were kept at 4°C overnight. An extract of 1mL of supernatant was taken and mixed with 1mL 2NH_2_SO_4_. From the mixture,0.5 ml was further mixed with.5 ml of KI_3_. The mixture was cold at 90 min, then, was added a pre-chilled distilled water and 1,2-dichloroethane. It will form two layers, the upper layer will be discarded, and absorbance was taken from the spectrophotometer at 365 nm. Endogenous ascorbic acid was determined according to the standard protocol (Mukherjee and Choudhuri, 1983). Fresh leaf material (0.1 g) of each wheat replicate and treatment was extracted with 5 ml of 6% trichloroacetic acid. The extract was centrifuged at 12,000 rpm for 15 min. Two milliliters of the extract was thoroughly mixed with 1 mL of 2% dinitrophenyl hydrazine (in acidic medium), followed by the addition of one drop of 10% thiourea (in 70% ethanol). The mixture was placed in a water bath for 15 min and cooled at room temperature, then, 2.5 ml of 80% (v/v) H_2_SO_4_ was further added to the mixture at 0°C. The absorbance was read at 530 nm with a spectrophotometer.

The proline contents were measured using the protocol given by Bates et al. (1973). Briefly, 2 ml proline extract, 2 ml of glacial acetic acid, and 2 ml of acid ninhydrin were mixed, placed (1 h) in a boiling water bath, and finally put in an ice bath. Later, the absorbance (520 nm) of the reaction mixture was noticed on the Spekol Spectrocololourimeter, VEB Carl Zeiss. To estimate the final proline contents, a standard curve was drawn by using a known concentration of authentic proline. Tocopherol contents were estimated by the methodology devised by Backer et al. (1980). Fresh leaf (1 g) was homogenized with a mixture of petroleum ether and ethanol (2:1.6 v/v). The extract was centrifuged at 10,000 rpm for 20 min to gain clear supernatant. One milliliter of extract and 2% of 2,2-dipyridylethnolic solution was mixed, then the mixture was kept in dark for 5 min. The resulting color in the aqueous layer was used to estimate tocopherol at 520 nm.

### Determination of Flavonoids, Total Free Amino Acid, Phenolics, and Total Soluble Sugars

Flavonoid contents were determined by following the method of Kim et al. (1999). Fresh leaves (0.2 g) were ground in 80% acetone and plant extract was filtered. Later, 1 ml of plant extract was mixed in a 10 ml volumetric flask having 4 ml distilled water. The further reaction mixture was mixed with 5% NaNO_3_, 10% AlCl_3_, and 1M NaOH with a break of 5 and 2 min, respectively. The reaction mixture was diluted with 3mL of distilled water. The absorbance was taken at 510 nm. The quercetin is used as standard. Hamilton and Van Slyke’s (1943) method, with a slight modification of quantities to estimate total free amino acid in treated and non-treated wheat plant samples. Enzyme extract (0.5 ml) was mixed with a solution containing 10% pyridine (0.5 ml) and 20% ninhydrin solutions (0.5 ml). The reaction mixture was heated in a water bath by covering the tubes with aluminum foil at 50°C for 30 min; the volume is up to 25 ml with distilled water. Optical density was taken at 570 nm. Phosphate buffer is used as blank. Total soluble phenolic was analyzed according to Julkunen-Titto (1985). A known weight of fresh leaf sample was homogenized with 80% acetone followed by centrifugation for 15 min. at 1,000 g. Then, supernatant (0.1 ml) was mixed with 2 ml of water and 1 ml of Folin-Ciocalteau’s phenol reagent and was shaken well. About 5 ml of 20% Na_2_CO_3_ was added with 10 ml of distilled water. Optical density (OD) was taken with a spectrophotometer (IRMECO 2020) at 750 nm. Total soluble sugar (TSS) contents were extracted by using fresh leaves (0.1 g) of both cultivars by cutting and boiling them in the distilled water (5 ml) for 1 h. The extract was filtered and the volume was up to 25 ml. The TSS was analyzed by using a reaction of 1 ml of an extract with 5 ml fresh anthrone reagent (150 mg anthrone, + 100 ml 72% H_2_SO_4_) (Handle, 1968). Thereafter, the reaction mixture was placed in a boiling water bath for 10 min. The absorbance of the sample and blank was determined at 620 nm using IRMECO 2020.

### Determination of Cd Contents

The root and leaf samples were dried in a hot-air oven at 72°C for six days. Shoot and root dry samples (0.25 g) were separately placed in a 50 ml digestion flask and digested in 5 ml concentrated nitric acid (HNO_3_) and kept at room temperature for 3 h. The samples were transferred to a heating block and digested at 300°C until samples turned black to tinted yellow. The samples were taken from the heating block, cooled, diluted with distilled water up to 25 ml, and were filtered. The acid extract of the samples was used to determine Cd concentration by atomic absorption spectrum (PerkinElmer, Waltham, MA, United States).

### Statistics Analysis

All the attributes discussed above were measured by 45 DAS and subjected to statistical analysis using the SPSS (SPSS 21.0 statistical software package). For each treatment, the mean values with SE (± SE) are described in the figures and tables. The parameters were analyzed by multivariate ANOVA (MANOVA). The significant difference among the means was calculated by Tukey’s test (*p* < 0.05).

## Results

### Growth Responses With Foliar Spray of Bio-Stimulants Under Cd Stress

All growth attributes, such as shoot length, roots length, the number of tillers per plant, the number of leaves per plant, and leaf area, were significantly reduced under Cd stress, while significantly increased with foliar spray of MLE and AsA, except root length (Table 1). Compared to the control (NS) plants (0 μM Cd), the shoot length of the Fsd-08 cultivar was reduced by 29 and 46% under 500 μM and 1000 μM Cd stress, respectively. Likewise, at the highest Cd concentration (1,000 μM), the leaf area, the number of leaves per plant, and the number of tillers per plant were reduced by 45 and 36%, 48 and 57%, and 71 and 75% in Fsd-08 and Glxy-13 cultivars, respectively (Table 1). However, exogenous application of AsA and MLE significantly improved (particularly at 500 μM Cd stress) growth traits except for root length. In comparison to the stressed plants (500 μM Cd), exogenous application of MLE exhibited a prominent improvement in shoot length (24 and 30%), leaf area (30 and 63%), number of tillers per plant (23% and 34%), and number of leaves per plant (42 and 31%) in Fsd-08 and Glxy-13 cultivars, respectively.

**TABLE 1:** Variation among morphological attributes under the foliar spray of ascorbic acid (AsA) and moringa leaf extract (MLE), along with no-spray (NS) in two wheat cultivars (Fsd-08 and Glxy-13) exposed to the cadmium (Cd) toxicity.

Amendment	Cd (μM)	Shoot Length (cm)		Root Length (cm)		Leaf area (cm^2^)		No. of tillers (Per plant)		No. of leaves (Per plant)	
		Fsd-08	Glxy-13	Fsd-08	Glxy-13	Fsd-08	Glxy-13	Fsd-08	Glxy-13	Fsd-08	Glxy-13
NS	0	42.7 ± 2.4 b	40.5 ± 3.3 b	18.75 ± 2.2 b	16.5 ± 1.3 b	28.6 ± 3.1 a	16.6 ± 3.1 b	4.5 ± 0.6 b	4.0 ± 0.8 b	11.0 ± 0.8 b	11.5 ± 0.6 ab
	500	30.3 ± 1.7 d	26.2 ± 2.06 d	12.0 ± 2.3 c	12.5 ± 3.0 d	15.1 ± 0.6 c	14.1 ± 0.3 c	3.5 ± 0.6 b	3.2 ± 0.5 c	7.6 ± 0.9 c	8.8 ± 0.5 c
	1000	23.0 ± 2.9 e	23.0 ± 1.8 e	7.5 ± 0.6 d	7.5 ± 0.5 e	15.8 ± 1.9 c	10.6 ± 0.6 d	1.3 ± 0.5 d	1.0 ± 0.0 e	5.7 ± 0.9 c	5.0 ± 1.4 d
AsA	0	45.5 ± 3.5 a	42.7 ± 1.7 a	19.3 ± 1.97 a	20.0 ± 1.1 a	29.9 ± 2.0 a	20.4 ± 1.9 a	5.5 ± 1.0 a	4.8 ± 0.5 a	13.2 ± 0.9 a	12.6 ± 0.9 a
	500	35.0 ± 2.8 c	35.7 ± 4.1 c	12.5 ± 1.7 c	13.5 ± 2.4 cd	18.3 ± 0.9 b	14.4 ± 1.0 c	4.0 ± 1.1 b	3.8 ± 1.0 c	9.5 ± 0.5 b	11.0 ± 0.8 b
	1000	23.3 ± 1.0 e	26.0 ± 2.4	7.2 ± 1.7 d	6.8 ± 0.5 e	18.3 ± 1.1 b	11.6 ± 1.5 d	2.5 ± 0.6 c	2.3 ± 0.5 d	6.0 ± 1.2 c	5.7 ± 0.9 d
MLE	0	45.5 ± 4.0 a	45.5 ± 4.0 a	20.8 ± 2.8 a	21.4 ± 0.5 a	29.7 ± 3.6 a	22.5 ± 5.9 a	5.5 ± 0.6 a	5.5 ± 0.5 a	14.0 ± 0.8 a	13.3 ± 1.3 a
	500	37.5 ± 2.5 c	34.0 ± 4.6 c	13.0 ± 2.2 c	14.0 ± 1.6 c	19.6 ± 1.1 b	17.3 ± 3.2 b	4.3 ± 0.6 b	4.3 ± 0.5 b	10.8 ± 0.5 b	11.5 ± 0.6 ab
	1000	30.5 ± 3.0 d	28.7 ± 3.4 d	6.75 ± 1.3 d	7.3 ± 0.9 e	14.1 ± 2.9 c	14.3 ± 3.7 c	1.7 ± 0.5 d	2.0 ± 0.0 d	6.0 ± 1.4 c	6.0 ± 0.0 d

*The means sharing different lowercase letters differ significantly as determined by LSD test at p ≤ 0.05. The data presented is average of 4 replications ± SD. AsA and MLE were applied at the rate of (50 mM) and (3% w/v), respectively.*

### Gas Exchange Responses With Foliar Spray of Bio-Stimulants Under Cd Stress

The results exhibited that carbon dioxide assimilation rate (*A*), transpiration rate (*E*), water use efficiency (*WUE*), and stomatal conductance (*G*_*s*_) in both wheat cultivars were deprived of the increasing levels of Cd stress, except internal CO_2_ concentration (*Ci*) (Table 2). However, a highly significant impact of MLE and AsA was recorded to mitigate Cd stress up to 500 μM for all gaseous attributes. The CO_2_ assimilation rate (*A*) was prominently increased with the application of both foliar stimulants, with and without Cd stress (Table 2). Both wheat cultivars showed a significant decline in transpiration rate € under 1,000 μM Cd toxicity, as compared to the non-stressed seedlings. However, the transpiration rate was maintained to normal with foliar spray of MLE and AsA under the lower Cd stress (500 μM). The water-use efficiency (WUE), though depressed with Cd stress in both wheat cultivars, was significantly enhanced after the foliage application of MLE and AsA. Overall, the Fsd-08 cultivar showed greater gaseous exchangeability compared with the Glxy-13 cultivar. The stomatal conductance of both wheat cultivars showed a non-significant difference in all the treatments. The intercellular CO_2_ concentration (*Ci*) was increased under stress, while slightly decreased by MLE and AsA foliar treatments (Table 2).

**TABLE 2:** Variation among gaseous exchange attributes under the foliar spray of AsA and MLE, along with NS in two wheat cultivars (Fsd-08 and Glxy-13) exposed to the Cd toxicity.

Amendment	Cd	CO_2_ assimilation		Net transpiration		Water use		Stomatal		Internal CO_2_	
	(μM)	rate (A)		rate (E)		efficiency (WUE)		conductance (Gs)		concentration (Ci)	
		(mmol CO_2_ m^–2^ s^–1^)		(mmol H_2_O m^–2^ s^–1^)		(mmol CO_2_/mmol H_2_O)		(nmol CO_2_ m^–2^ s^–1^)		(μmol CO_2_ m^–2^ s^–1^)	
		Fsd-08	Glxy-13	Fsd-08	Glxy-13	Fsd-08	Glxy-13	Fsd-08	Glxy-13	Fsd-08	Glxy-13
NS	0	10.4 ± 0.5 c	9.7 ± 0.4 c	1.34 ± 0.04 b	1.37 ± 0.51 b	7.78 ± 0.43 d	7.72 ± 2.12 b	0.08 ± 0.01 b	0.09 ± 0.01 b	205.0 ± 5.7 d	203.3 ± 14.4 d
	500	7.4 ± 0.5 d	8.0 ± 0.1 d	1.63 ± 0.31 a	1.83 ± 0.09 a	4.66 ± 0.97 e	4.38 ± 0.18 c	0.07 ± 0.01 b	0.05 ± 0.01 b	225.8 ± 10.1 c	230.2 ± 10.5 c
	1000	5.7 ± 0.3 e	4.5 ± 0.4 e	0.78 ± 0.17 d	0.67 ± 0.09 d	1.98 ± 0.14 f	1.67 ± 0.08 d	0.08 ± 0.02 b	0.06 ± 0.01 b	324.6 ± 18.2 a	305.3 ± 17.8 a
AsA	0	13.9 ± 0.9 b	10.7 ± 1.1 b	1.28 ± 0.22	1.40 ± 0.53 b	11.03 ± 1.64 b	8.25 ± 2.24 a	0.11 ± 0.01 a	0.10 ± 0.02 a	186.4 ± 17.3 e	172.2 ± 30.8 f
	500	12.9 ± 0.6 b	11.2 ± 0.8 b	1.48 ± 0.53 b	1.22 ± 0.16 c	9.79 ± 3.89 c	9.29 ± 1.13 a	0.09 ± 0.01 b	0.07 ± 0.02 b	232.2 ± 14.3 c	229.5 ± 9.3 c
	1000	10.2 ± 1.2 c	10.3 ± 0.8 bc	1.04 ± 0.49 c	0.63 ± 0.23 d	4.62 ± 1.16	4.52 ± 1.39 c	0.08 ± 0.01 b	0.06 ± 0.01 b	284.7 ± 10.4 b	246.8 ± 1.72 b
MLE	0	14.2 ± 0.4 a	10.9 ± 0.6 b	1.10 ± 0.05 c	1.37 ± 0.33 b	12.91 ± 0.86 a	8.38 ± 2.20 a	0.12 ± 0.02 a	0.10 ± 0.02 a	189.1 ± 19.5 e	193.7 ± 17.3 e
	500	13.9 ± 1.6 b	12.2 ± 0.2.7 a	1.09 ± 0.05 c	1.65 ± 0.26 a	12.81 ± 1.48 a	7.44 ± 1.28 b	0.09 ± 0.02 b	0.07 ± 0.01 b	241.3 ± 6.6 c	221.4 ± 5.7 c
	1000	10.7 ± 1.3 c	9.7 ± 0.4 c	0.67 ± 0.04 d	0.55 ± 0.08 d	4.53 ± 0.69 e	4.44 ± 1.29 c	0.08 ± 0.01 b	0.06 ± 0.01 b	269.1 ± 5.5 b	257.9 ± 7.4 b

*The means sharing different lowercase letters differ significantly as determined by LSD test at p ≤ 0.05. The data presented is average of 4 replications ± SD. AsA and MLE were applied at the rate of (50 mM) and (3% w/v), respectively.*

### Biomass Accumulation and Photosynthetic Pigments With Foliar Spray of Bio-Stimulants Under Cd Stress

**FIGURE 1:**
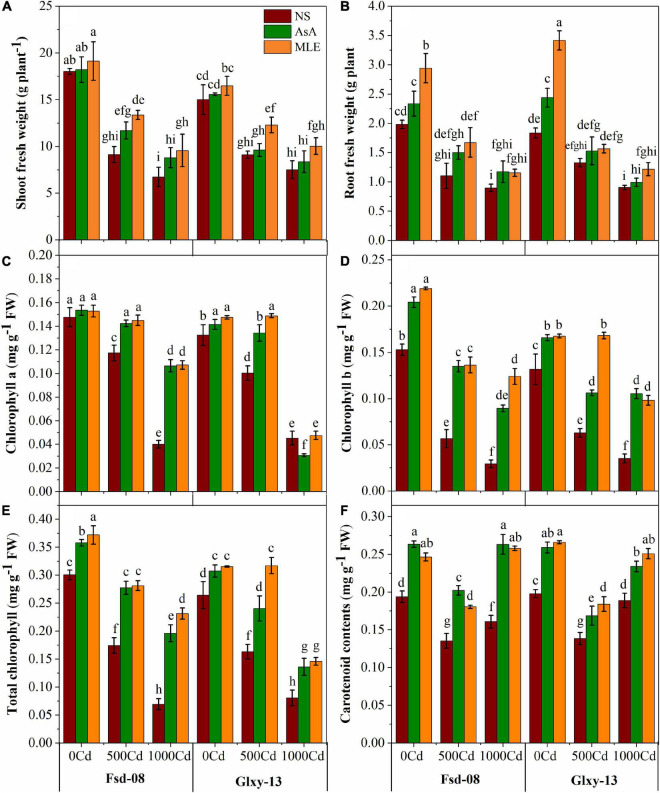
Fresh biomass and photosynthetic pigment responses as **(A)** shoot fresh weight, **(B)** root fresh weight, **(C)** chlorophyll a, **(D)** chlorophyll b, **(E)** total chlorophyll, and **(F)** carotenoid contents, under the foliar spray of ascorbic acid (AsA) and moringa leaf extract (MLE), along with no-spray (NS) in two wheat cultivars (Fsd-08 and Glxy-13) exposed to the cadmium (Cd) toxicity. Different lowercase letters denote significant difference as determined by least significant difference (LSD) test (*p* < 0.05, *n* = 4). AsA and MLE were applied at the rate of 50 mM and 3% (w/v), respectively.

A reduction in biomass accumulation and photosynthetic attributes governed by Cd stress was concentration-dependent; therefore, higher levels of Cd resulted in a higher loss in biomass accumulation and chlorophyll contents in both wheat cultivars (Figure 1). The results exhibited that Cd toxicity significantly reduced the shoot and root fresh biomass of both wheat cultivars (Fsd-08 and Glxy-13). However, the application of MLE has highly improved the biomass accumulation (46 and 35%) and (52 and 18%) under the lower Cd stress (1,000 μM), respectively, compared to the relevant treatment without MLE application. Total chlorophyll contents were decreased by 76 and 69% in Fsd-08 and Glxy-13 under Cd stress (1000 μM), compared with normal growth conditions, respectively. However, this loss was minimized with the foliar application of MLE and AsA. Moreover, carotenoid contents increased in a parallel way with increasing Cd stress by utilizing both sources of foliar applications (Figure 1).

### Chlorophyll Fluorescence Responses With Foliar Spray of Bio-Stimulants Under Cd Stress

**FIGURE 2:**
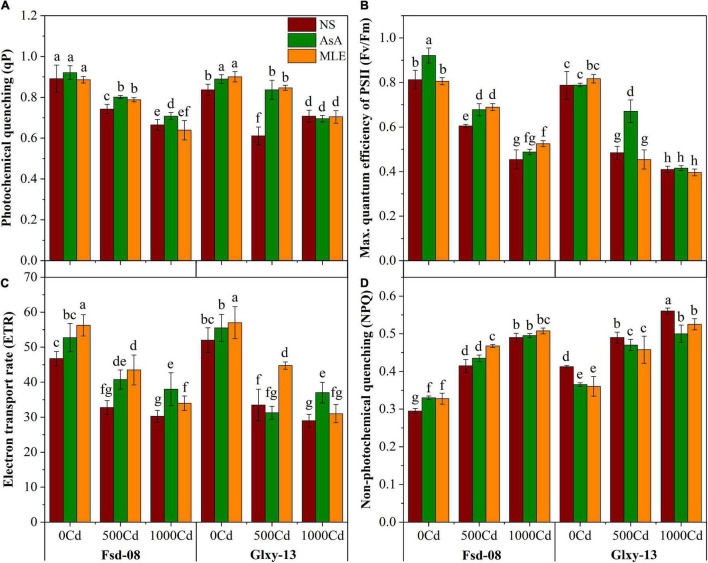
Chlorophyll fluorescence responses as **(A)** photochemical quenching (qP), **(B)** maximum quantum efficiency of PSII (Fv/Fm), **(C)** electron transport rate (ETR), and **(D)** non-photochemical quenching (NPQ), under the foliar spray of AsA and MLE, along with N) in two wheat cultivars (Fsd-08 and Glxy-13) exposed to the Cd toxicity. Different lowercase letters denote significant difference as determined by LSD test (*p* < 0.05, *n* = 4). AsA and MLE were applied at the rate of 50 mM and 3% (w/v), respectively.

A significant difference in chlorophyll fluorescence traits was recorded among all the treatments that are applied on the wheat cultivars (Figure 2). Photochemical quenching (qP), the maximum quantum efficiency of photosystem II (*F*_*v*_/*F*_*m*_), and electron transport rate have significantly declined with the increasing Cd stress in the rhizosphere, with one exception of non-photochemical quenching (NPQ), which increased significantly under the elevated level of Cd concentration in both wheat cultivars (Figure 2). Wheat seedlings grown under 1,000 μM Cd concentration showed relatively higher NPQ values compared to 500 μM Cd alone and in combination with AsA or MLE (Figure 2D). However, foliar application of AsA and MLE significantly enhanced the qP, *Fv/*Fm, and ETR values under 500 μM Cd stress, compared to the non-sprayed stressed seedlings of both wheat cultivars (Figures 2A–C).

### Osmolytes Regulation in Wheat Leaves With Foliar Spray of Bio-Stimulants Under Cd Stress

**FIGURE 3:**
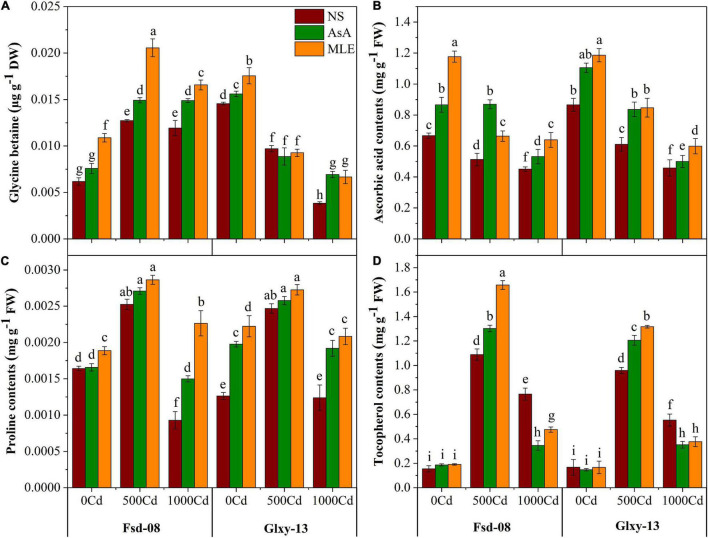
Osmolyte accumulation responses as **(A)** glycine betaine, **(B)** AsA, **(C)** proline contents, and **(D)** tocopherol contents, under the foliar spray of AsA and MLE, along with NS in two wheat cultivars (Fsd-08 and Glxy-13) exposed to the Cd toxicity. Different lowercase letters denote significant difference as determined by LSD test (*p* < 0.05, *n* = 4). AsA and MLE were applied at the rate of 50 mM and 3% (w/v), respectively.

According to the obtained results, ascorbic acid contents have decreased, while tocopherol, glycine betaine, and proline contents have increased under the Cd toxicity, particularly with 500 μM Cd concentration. Meanwhile, exogenous application of biostimulants modulated the innate mechanisms to boost the non-enzymatic antioxidant defense system of both wheat cultivars (Figure 3). Briefly, glycine betaine (GB) contents were enhanced significantly after applying MLE (62%) and AsA (17%) under Cd stress (500 μM) in Fsd-08 cultivar, compared to the corresponding treatments without MLE spray. However, Fsd-08 accumulated higher GB contents compared to the Glxy-13 under the Cd stress in combination with MLE or AsA. Furthermore, the experimental data expressed that AsA concentration in the leaves of Fsd-08 was decreased by 23 and 32% under 500 and 1,000 μM Cd stress, respectively, compared to the control. Meanwhile, foliar application of AsA mitigated the Cd stress (500 μM) by elevating *in vivo* the ascorbic acid pool by 70 and 37% in Fsd-08 and Glxy-13, respectively, compared with the relevant treatments without AsA application. Proline contents varied significantly (*p* ≤ 0.05), with and without a foliar spray of MLE and AsA, in both cultivars exposed to Cd stress (Figure 3). The alpha-tocopherol contents in wheat cultivars also exhibited a significant (*p* ≤ 0.05) escalation with the exposure to Cd stress. However, the effect of MLE and AsA on alpha-tocopherol contents was more obvious at 500 μM Cd stress compared to the 1,000 μM Cd level. After the supply of MLE and AsA, an increment in tocopherol contents was noticed by 52 and 20% in Fsd-08, while 37 and 26% in Glxy-13 cultivar exposed to 500 μM Cd stress, respectively, compared with the related treatments without a foliar spray of biostimulants.

### Modulation in Biological Compounds With Foliar Spray of Bio-Stimulants Under Cd Stress

**FIGURE 4:**
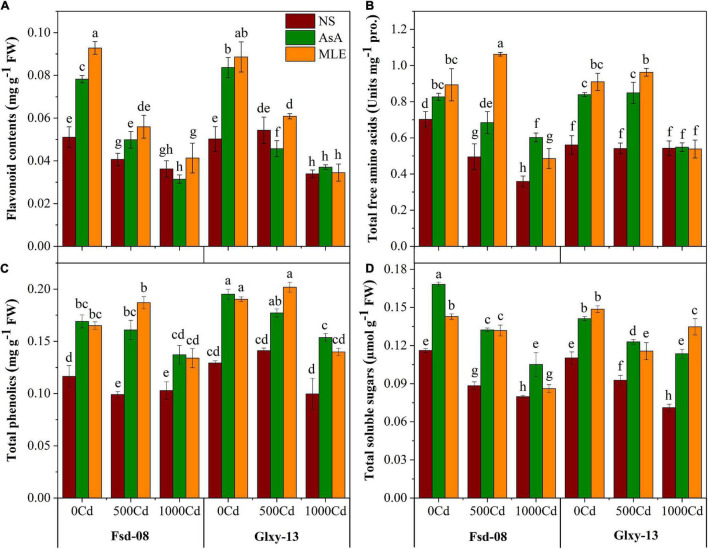
Osmolyte accumulation responses as **(A)** flavonoid contents, **(B)** total free amino acid, total free amino acid (TFAA), **(C)** total phenolics, and **(D)** total soluble sugars, under the foliar spray of AsA and MLE, along with NS in two wheat cultivars (Fsd-08 and Glxy-13) exposed to the Cd toxicity. Different lowercase letters denote the significant difference as determined by LSD test (*p* < 0.05, *n* = 4). AsA and MLE were applied at the rate of 50 mM and 3% (w/v), respectively.

Various biological compounds, such as flavonoids, total free amino acid (TFAA), total phenolics, and TSS contents, decreased with the increasing Cd stress (Figure 4). Both wheat cultivars showed a significant (*p* ≤ 0.05) reduction for flavonoid pool and total free amino acids at all levels of Cd stress. However, flavonoid contents displayed a positive response in Fsd-08 and Glxy-13 cultivars after the foliar spray of MLE (38% and 12%), in reaction with 500 μM Cd stress, respectively, relative to the similar treatments without MLE application. However, the mitigation of 1,000 μM Cd concentration was not obvious with the spraying of biostimulants and of flavonoid contents that are sharply diminished (Figure 4A). The seedlings exposed to Cd showed severe reduction in TFAA contents up to 41–95% and 25–29% in Fsd-08 and Glxy-13, respectively, compared to the NS (no-spray) treatment. However, foliar spray of AsA and MLE alleviated the Cd (500 μM) stress and produced more TFAA contents (38% and 114%) in Fsd-08 cultivar, respectively, compared to the relevant treatment without biostimulants application (Figure 4B). Similarly, phenolic contents were declined drastically by 12% in Fsd-08 and 64% in Glxy-13 with the highest level of Cd (1,000 μM), compared to the control plants. However, in MLE-treated plants, phenolic activity was enhanced up to 30% and 40% in the leaves of Fsd-08 and Glxy-13 grown under 1,000 μM Cd stress, respectively. The application of MLE and AsA showed a better response under 500 μM Cd stress but AsA presented a slightly better behavior by elevating phenolic contents even under 1000 μM Cd stress in both wheat cultivars (Figure 4C). Likewise, TSS contents were also depressed due to the Cd stress in both wheat cultivars. On contrary to the negative effects of Cd, MLE application substantially increased the contents of TSS in the leaf tissues of wheat. Briefly, Cd tends to decrease TSS contents by 31–63%; however, foliar spray of biostimulants recovered its status up to 20–25%, compared to the no-spray (NS) plants (Figure 4D).

### Cd Concentration Modulation in Wheat With Foliar Spray of Bio-Stimulants Under Cd Stress

**FIGURE 5:**
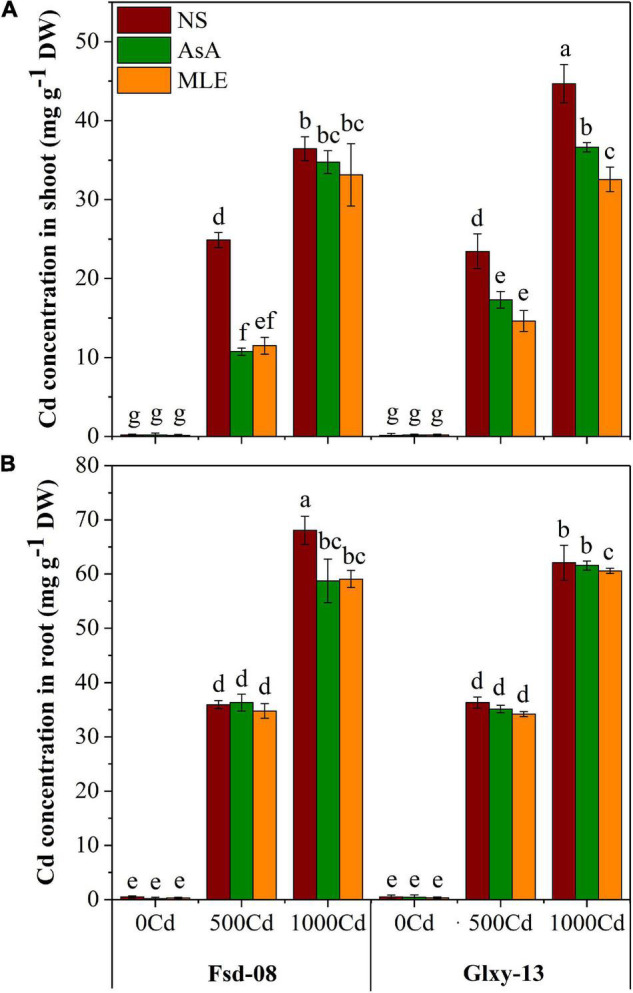
Accumulation of Cd in shoot **(A)** and root **(B)**, under the foliar spray of AsA and MLE, along with NS in two wheat cultivars (Fsd-08 and Glxy-13) exposed to the Cd toxicity. Different lowercase letters denote the significant difference as determined by LSD test (*p* < 0.05, *n* = 4). AsA and MLE were applied at the rate of 50 mM and 3% (w/v), respectively.

The Cd concentrations in the different wheat tissues (shoot and root) under the foliar applications of the two biostimulants on two wheat cultivars are presented in Figure 5. The foliar application of AsA and MLE significantly (*p* < 0.05) reduced Cd accumulation in the shoot in root tissues of both wheat cultivars in comparison to the no-spray treatment. Cd concentration in the shoot reduced by 58–61% in Fsd-08 and 33–40% in Glxy-13 with the foliar spray of AsA followed by MLE under the lower Cd stress (500 μM).

### Relationship of Cd Accumulation in Wheat Tissues With Measured Attributes of Wheat

**FIGURE 6:**
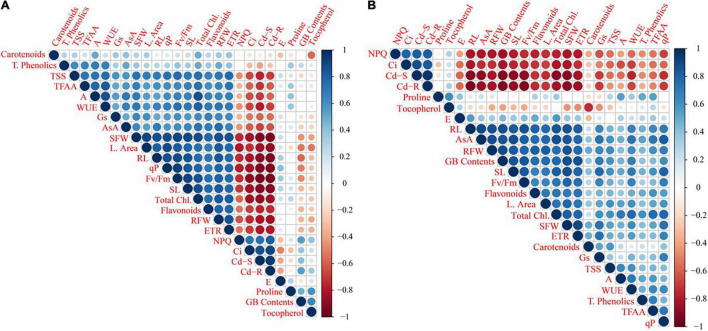
The relative correlation analysis among growth and physio-biochemical attributes of two wheat cultivars, i.e., Fsd-08 **(A)** and Glxy-13 **(B)**, under the foliar spray of AsA and MLE, along with NS treatments. The blue and red colors indicate positive (+) and negative (-) correlation, whereas size of circle and color deepness indicates the intensity (stronger or weaker) of correlations at *P* < 0.05, respectively. The abbreviations are as following: qP: photochemical quenching; TFAA: total free amino acid contents; WUE: water use efficiency; TSS: total soluble sugars; ETR: electron transport rate; SFW: shoot fresh weight; RFW: root fresh weight; Fv/Fm: max. quantum efficiency of PSII; GB: glycine betaine contents; NPQ: non-photochemical quenching; RL: root length; SL: shoot length; AsA: ascorbic acid contents.

A correlation analysis was performed to evaluate the linkage between the accumulated Cd concentration and various physio-biochemical attributes of Cd-stressed wheat cultivars after the amendment of MLE and AsA (Figure 6). There is a significantly positive relationship of Cd contents in root and shoot tissues, with intercellular CO_2_ concentration (Ci) and non-photochemical quenching (NPQ), while a negative interaction was observed with other attributes of both wheat cultivars.

## Discussion

Minimization of Cd uptake in the wheat plant is key to decreasing the Cd toxicity, which ultimately ensures food safety (Ali et al., 2015; Wu et al., 2020). Crop health and vigor are addressed by its growth attributes. The detrimental effects of various heavy metals including Cd may be attributed to the modulation of the plant’s morpho-physiological and biochemical pathways, which indirectly influence plant growth (Kamran et al., 2019b; Turan, 2020, 2021b). It has been well-established that heavy metal contamination may cause a reduction in plant growth and physiological characteristics (Ali et al., 2014a; Kamran et al., 2019a). On the other hand, AsA application could be involved in improving the essential nutrients, which are the key growth-limiting elements to mitigate the hostile effects of different ecological stresses (Arshad et al., 2016; Golubkina et al., 2019). The MLE is rich in zeatin and other growth-enhancing substances, which are directly involved in improving the growth-related features of wheat (Barciszewski et al., 2000). According to the previous findings, the use of MLE *via* foliage has already been proven to boost plant growth (Maishanu et al., 2017). Moreover, MLE blended with growth-promoted substances, enhances germination parameters and seedling performance in plants (Khan et al., 2017). The chlorophyll fluorescence attributes are useful indicators for growth, physiological response, and modulation of PSII in plants under abiotic and biotic stress. The current data suggested a direct relationship between the growth and the fluorescence parameters because the growth and chlorophyll fluorescence attributes were affected by all Cd levels (Figures 1, 2). According to the findings of Yotsova et al. (2020), the reduced chlorophyll contents displayed an impaired PSII reaction center. A decrease in *qP* value may be attributed to the closed stomatal apertures and restrict the conversion of light energy into chemical energy (Gao et al., 2021), which was also evident from the significant reduction in *Fv/Fm* values (Figure 2B). Moreover, the application of AsA mitigated the adverse effects of Cd by enhancing the photoprotection ability. However, Plumb et al. (2018) reported that AsA is an essential growth-promoting substance, but is not involved in the photoprotection of *Arabidopsis thaliana*.

It was previously exhibited that Cd stress downregulated the gas exchange features in wheat seedlings (Khan et al., 2017). Moreover, Cd caused a considerable loss in photosynthetic pigments by degrading the chlorophyll apparatus (Ali et al., 2014b; Yan et al., 2021). The suppression in photosynthetic attributes, *i.e.*, decreased chlorophyll contents, gas exchange, and transpiration due to Cd, emerges as the main cause for the decline in growth and other associated characteristics (Aqeel et al., 2021; Rahman et al., 2021). According to Shah et al. (2019), wheat seeds treated with ASA maximized chlorophyll contents, tillers per plant, number of grains per spike, and 1,000-grain weight. This finding further elaborated that AsA could persuade the upregulation of SOD, POD, and CAT activities, thus, offsetting the adversities on wheat. The increase in chlorophyll contents may also depict a shielding role of AsA on the photochemical efficiency, which influenced the photochemical reactions. Our results conform with those related to the downturn of photochemical efficiency with Cd toxicity, and projection in photosynthetic attributes with the foliar application of AsA (Gaafar et al., 2020). The higher effectiveness of MLE for photosynthetic attributes in the recent trial is likely to be coupled with various allelochemicals and secondary metabolites, such as ascorbate, phenols, and zeatin, which are naturally present in the leaves of moringa (Foidl et al., 2001). Moreover, improvement in CO_2_ assimilation rate and *Gs* due to MLE is a consequence of essential metabolites and antioxidants that are present in the moringa leaves (Mona, 2013). Similar findings have previously suggested that MLE was found to be a potential amplifier for growth attributes, chlorophyll *a* and *b*, stomatal conductance (*Gs*), total soluble protein, and ascorbic acid in the rocket plant (*Erusa vesicaria*) (Abdalla, 2012).

To rescue crop plants from oxidative injury caused by abiotic stresses, the antioxidant enzymes and other self-defense systems display an important role (Kamran et al., 2020, 2021). It has been suggested that the increment in Cd contamination can lead to increased levels of oxidative damage (H_2_O_2_), ionic leakage, and lipid peroxidation resulting in an escalation in the activities of non-enzymatic antioxidants, antioxidative enzymes, and enzyme gene expression (Alharby et al., 2021). Cd stress also mediates the osmolyte accumulation in various plants (Zhao et al., 2013). The accumulation and biosynthesis of bioactive, physiologically active, and low molecular weight osmolytes serve as defensive molecules to avoid protein decay and breakage of cell structures without hampering the normal metabolic activities in the plant (Dumanović et al., 2021). Different plant species, as well as compartments of the cells, have been specialized to synthesize varying concentrations of such organic molecules under environmental stresses (Lugan et al., 2010). It has also been demonstrated that upregulation of amino acid metabolism, alkaloid biosynthesis, and proline metabolism. Furthermore, various metabolic intermediates are involved in the biosynthesis of the antioxidant defense system, cell wall, and phytochelatin metabolism allied with other organic ligands, playing vital roles in creating the Cd tolerance in plants (Lu et al., 2021). It was reported that foliar spray of AsA enhanced the peroxidase (POD) activity, as well as the phenolic contents and under stressed conditions (Gaafar et al., 2020). Currently, it was reported that moringa leaves contain a varying concentration of phytohormones, associated with seasonal changes of temperature of day length (Brockman et al., 2020). This attribute of moringa leaves makes them ideal to utilize its extract against abiotic stresses in wheat and other plants.

The cascade of AsA biosynthesis under the heavy metal stress depends on the length of its exposure period in the plant and its sole properties. The accumulation of free AsA in plant cells is of enormous significance for scavenging ROS, which could also be involved in the activation of both non-enzymatic and enzymatic antioxidant systems (Xu et al., 2015). Ascorbic acid is the most vital antioxidant in moringa, having a super-active role in enduring non-biological stress conditions. Previous studies have confirmed that ascorbic acid contents in various tissues of *Malus domestica* (Bai et al., 2013) and *Brassica napus* L. (Naeem et al., 2012) have been increased when treated with *Moringa oleifera* extract under abiotic stresses. Along with ascorbic acid, some other osmoprotectants like glycine betaine (GB) have been reported earlier in participating in osmotic balance and maintenance of macromolecules concentration, hence, improving the tolerance to abiotic conditions (Ahanger et al., 2017). Ascorbic acid acts as a precursor by restoring α-tocopherol and zeaxanthin levels in the xanthophyll cycle (Dumanović et al., 2021). Meanwhile, AsA also serves as a cofactor that adjusts the mode of action of many enzymes through a synergistic activation (Foyer and Noctor, 2005). Proline is a well-recognized aliphatic, a cyclized amino acid that plays a significant role to detoxify Cd stress. Proline concentration synergistically increased with phenylalanine biosynthesis for activating defense signaling cascades (Maeda and Dudareva, 2012). Furthermore, MLE contains many phytohormones such as cytokinins and indole-3-acetic acid (Vieira Santos et al., 2001). Under the non-biological stresses, the accumulation of sugars such as mannitol and galactinol takes place and confers tolerance in transgenic plants (Anjum et al., 2014). In addition, total soluble protein concentration and antioxidant activities were upgraded, thus, counteracting Cd stress in maize (Golubkina et al., 2019). A significant increase was reported in the emergence of the potential phenolics accumulation in the maize seedlings when the seeds were pre-treated with MLE (Basra et al., 2011), which might be due to the higher contents of vitamin C in MLE (Moyo et al., 2011). Cd accumulation in shoot and root tissues was increased with increasing Cd levels, as reported previously (Bashir et al., 2020). However, Cd accumulation was higher in roots than those in shoots. Similar findings were observed in our previous experiments (Fozia et al., 2018, 2021).

## Conclusion

**FIGURE 7:**
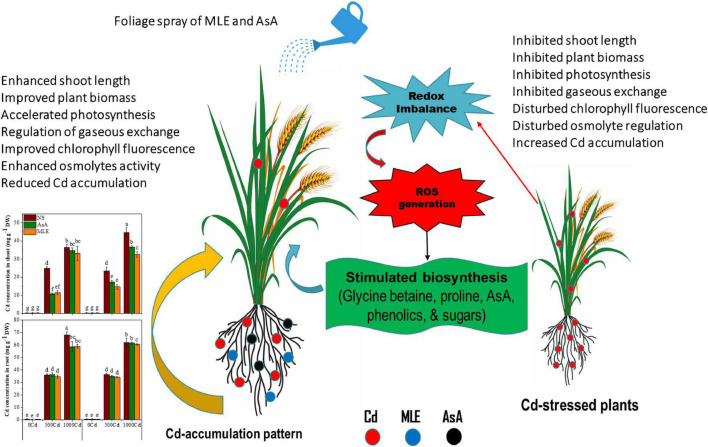
The schematic diagram exhibiting the beneficial role of AsA and MLE against Cd-induced phytotoxicity in wheat seedlings.

In this study, Cd caused a significant reduction in growth, photosynthetic apparatus, and osmoprotectant activities in two wheat cultivars, *i.e.*, Fsd-08 and Glxy-13. The mitigation of Cd toxicity by foliar spray of MLE was the most fascinating feature of the present study. An exogenous spray of MLE enhanced the phenolic, flavonoid, tocopherol, ascorbate, GB, proline, TSS, and TFAA, which directly or indirectly reciprocated morpho-physiological and biochemical parameters in contrast to non-spray plants under Cd toxicity. For AsA, in most of the studied features, MLE surpasses AsA in improving wheat performance under Cd stress. In a nutshell, an exogenous application of easily available and inexpensive natural bio-stimulants (especially MLE) could be a useful strategy to enhance Cd tolerance in wheat plants by improving growth, reducing Cd uptake, and accumulation of metabolites in various tissues (Figure 7).

## Data Availability Statement

The original contributions presented in the study are included in the article/supplementary material, further inquiries can be directed to the corresponding authors.

## Author Contributions

FF: data curation, formal analysis, investigation, and writing – original draft. MA: formal analysis, investigation, and writing – original draft. XW, RI, and HT: data curation and writing – review and editing. AT: supervision, writing – original draft, and writing – review and editing. MK: data curation, investigation, resources, writing – original draft, and writing – review and editing. IT: investigation and writing – review and editing. FM-P: data curation, investigation, and writing – review and editing. AE-S: resources and writing – review and editing. HE: resources, data curation, and writing – review and editing. All authors contributed to the article and approved the submitted version.

## Conflict of Interest

The authors declare that the research was conducted in the absence of any commercial or financial relationships that could be construed as a potential conflict of interest.

## Publisher’s Note

All claims expressed in this article are solely those of the authors and do not necessarily represent those of their affiliated organizations, or those of the publisher, the editors and the reviewers. Any product that may be evaluated in this article, or claim that may be made by its manufacturer, is not guaranteed or endorsed by the publisher.
