# Corrigendum: The Impact of Bio-Stimulants on Cd-Stressed Wheat (*Triticum aestivum* L.): Insights Into Growth, Chlorophyll Fluorescence, Cd Accumulation, and Osmolyte Regulation

**DOI:** 10.3389/fpls.2022.904893

**Published:** 2022-04-21

**Authors:** Fozia Farhat, Muhammad Arfan, Xiukang Wang, Arneeb Tariq, Muhammad Kamran, Hafiza Naila Tabassum, Ifra Tariq, Freddy Mora-Poblete, Rashid Iqbal, Ahmed M. El-Sabrout, Hosam O. Elansary

**Affiliations:** ^1^Department of Botany, University of Agriculture (UAF), Faisalabad, Pakistan; ^2^Department of Botany, Government College Women University, Faisalabad, Pakistan; ^3^Shaanxi Key Laboratory of Chinese Jujube, College of Life Sciences, Yan'an University, Yan'an, China; ^4^School of Agriculture, Food and Wine, The University of Adelaide, Adelaide, SA, Australia; ^5^Institute of Home and Food Sciences, Government College University Faisalabad, Faisalabad, Pakistan; ^6^Institute of Biological Sciences, University of Talca, Talca, Chile; ^7^Department of Agronomy, Faculty of Agriculture and Environment, The Islamia University of Bahawalpur, Bahawalpur, Pakistan; ^8^Department of Applied Entomology and Zoology, Faculty of Agriculture (EL-Shatby), Alexandria University, Alexandria, Egypt; ^9^Plant Production Department, College of Food & Agriculture Sciences, King Saud University, Riyadh, Saudi Arabia

**Keywords:** gaseous exchange rate, secondary metabolites, Cd accumulation, ascorbic acid, moringa leaf extract, growth, chlorophyll fluorescence

In the published article, there was an error in affiliation “Fozia Farhat^1,3^.” Instead of “Fozia Farhat^1,3^” it should be “Fozia Farhat^1,2^.”

The authors apologize for this error and state that this does not change the scientific conclusions of the article in any way. The original article has been updated.

## Publisher's Note

All claims expressed in this article are solely those of the authors and do not necessarily represent those of their affiliated organizations, or those of the publisher, the editors and the reviewers. Any product that may be evaluated in this article, or claim that may be made by its manufacturer, is not guaranteed or endorsed by the publisher.

